# DNAVaxDB: the first web-based DNA vaccine database and its data analysis

**DOI:** 10.1186/1471-2105-15-S4-S2

**Published:** 2014-03-19

**Authors:** Rebecca Racz, Xinna Li, Mukti Patel, Zuoshuang Xiang, Yongqun He

**Affiliations:** 1 Unit for Laboratory Animal Medicine, University of Michigan Medical School, Ann Arbor, MI 48109, USA; 2 College of Pharmacy, University of Michigan, Ann Arbor, MI 48109, USA; 3 College of Literature, Science, and the Arts, University of Michigan, Ann Arbor, MI 48109, USA; 4 Department of Microbiology and Immunology, University of Michigan Medical School, Ann Arbor, MI 48109, USA; 5 Center for Computational Medicine and Biology, University of Michigan, Ann Arbor, MI 48109, USA; 6 Comprehensive Cancer Center, University of Michigan Medical School, Ann Arbor, MI 48109, USA

**Keywords:** DNA vaccine, database, DNAVaxDB, protective antigen, plasmid, bioinformatics

## Abstract

Since the first DNA vaccine studies were done in the 1990s, thousands more studies have followed. Here we report the development and analysis of DNAVaxDB (http://www.violinet.org/dnavaxdb), the first publically available web-based DNA vaccine database that curates, stores, and analyzes experimentally verified DNA vaccines, DNA vaccine plasmid vectors, and protective antigens used in DNA vaccines. All data in DNAVaxDB are annotated from reliable resources, particularly peer-reviewed articles. Among over 140 DNA vaccine plasmids, some plasmids were more frequently used in one type of pathogen than others; for example, pCMVi-UB for G- bacterial DNA vaccines, and pCAGGS for viral DNA vaccines. Presently, over 400 DNA vaccines containing over 370 protective antigens from over 90 infectious and non-infectious diseases have been curated in DNAVaxDB. While extracellular and bacterial cell surface proteins and adhesin proteins were frequently used for DNA vaccine development, the majority of protective antigens used in *Chlamydophila *DNA vaccines are localized to the inner portion of the cell. The DNA vaccine priming, other vaccine boosting vaccination regimen has been widely used to induce protection against infection of different pathogens such as HIV. Parasitic and cancer DNA vaccines were also systematically analyzed. User-friendly web query and visualization interfaces are available in DNAVaxDB for interactive data search. To support data exchange, the information of DNA vaccines, plasmids, and protective antigens is stored in the Vaccine Ontology (VO). DNAVaxDB is targeted to become a timely and vital source of DNA vaccines and related data and facilitate advanced DNA vaccine research and development.

## Background

A DNA vaccine is a bacterial DNA plasmid constructed to express an encoded protein or peptide antigen(s), which is administered *in vivo *and able to induce preventive or therapeutic antigen-specific immune response against a specific disease or infection. The DNA plasmid must encode a promoter that is active in mammalian cells [1]. The first DNA vaccine studies were conducted in the 1990s, with the first successful experiment done in 1990 when a DNA plasmid expressing a protein was injected into mice and the protein was successfully synthesized *in vivo *[2]. Once expressed, the protective protein antigen encoded by a gene in a DNA vaccine can be degraded into peptides by antigen presenting cells. The protective immune peptides (*i.e*., epitopes) can then be transferred to the cell surface and activate T cells. If the DNA vaccine is taken up by muscle cells, the muscle cells can transfer the expressed protein to the antigen presenting cells to stimulate the T cells. DNA vaccines can also stimulate antibody responses through antigen recognition by B cells [1]. Compared to other types of vaccines (*e.g*., live attenuated or killed whole organism vaccines, and subunit vaccine), DNA vaccines are safe, easy to prepare and store, and cost effective. In addition, DNA vaccines allow for focused immunity on the antigen of interest and have the ability to induce natural, long-lasting, and varied immune responses *in vivo*.

Intensive efforts have been taken to study and use DNA vaccines. There have been over 55,000 articles indexed in PubMed and Google Scholar combined. Four DNA vaccines have been licensed for veterinary uses. Among them are two DNA vaccines against infectious diseases, including West Nile-Innovator (Fort Dodge Animal Health, Fort Dodge, IA, USA) for protection against horse West Nile virus (https://online.zoetis.com/US/EN/Products/Pages/West_Nile_Innovator.aspx), and Apex-IHN (Novartis Animal Health, Basel, Switzerland) against infectious haematopoietic necrosis virus in salmon (http://www.vical.com/products/infectious-disease-vaccines/Apex-IHN/default.aspx). Oncept (Merial, Lyon, France), another licensed DNA vaccine, is for protection against dog melanoma [3]. In addition, one DNA vaccine has been licensed for a gene therapy application in food pigs for increasing litter survival of breeding sows: LifeTide SW 5 (VGX Animal Health, Inc. The Woodlands, TX, USA) (http://www.vgxah.com/LifetideSW5.html). Although there is not a licensed human DNA vaccine on the market, many human clinical trials for DNA vaccine evaluation are currently being conducted. Researchers are continuing to investigate the mechanisms behind DNA vaccination and vaccine immunity.

While intensive research has been conducted and resulted in promising DNA vaccine candidates, there is no web-based central resource that allows the storage, annotation, comparison, and analysis of experimentally verified DNA vaccines. VIOLIN (http://www.violinet.org) is the first web-based comprehensive vaccine database and analysis system that targets vaccine research [4,5]. Currently, VIOLIN has included more than 3,200 vaccines for over 180 infectious diseases and many non-infectious diseases (*e.g*., cancers and arthritis). Each of the curated vaccines has been shown to induce significant protection against a disease in a natural host or at least one laboratory animal model. Many of the vaccines in VIOLIN are verified DNA vaccines. However, the general VIOLIN system does not include any specific information about DNA vaccine plasmids. It does not include tools to specifically query and analyze those protective antigens used in DNA vaccines. The DNA vaccine-specific plasmids and protective antigens may preserve unique patterns that would support further DNA vaccine research and development. A specific query system for DNA vaccines is not available either in the general system. The DNA vaccine research has become its own independent research and has its own community (for example, there is an active DNA Vaccines LinkedIn group (http://www.linkedin.com/groups/DNA-Vaccines-3077917). Therefore, there is a need to develop a DNA vaccine specific resource.

To address the many needs of the DNA vaccine community, we have developed DNAVaxDB (http://www.violinet.org/dnavaxdb), a web-based DNA vaccine database and analysis system. As a relatively independent program under the umbrella of the VIOLIN vaccine database and analysis system, DNAVaxDB includes the information for a large set of DNA vaccines, DNA vaccine plasmids, and protective antigens used in DNA vaccines. The analysis of these data reveals many unique patterns specific for DNA vaccines.

## Methods

### DNAVaxDB system and database design

DNAVaxDB was developed with a three-tier architecture built on two HP ProLiant DL380 G6 servers that run the Redhat Enterprise Linux (release 5.8) operating system. A query on DNAVaxDB can be submitted from the DNAVaxDB web user interface (presentation tier). The query is then processed using PHP/SQL (middle tier, application server based on Apache) against a relational database (MySQL version 5.5.28) (data tier, database server). Query results are then displayed in the web browser. Furthermore, DNAVaxDB curators and reviewers can submit and review curated vaccine and plasmid data through the VIOLIN data curation system. Two servers regularly back up each other's data. DNAVaxDB is one relatively independent program under the umbrella of the VIOLIN vaccine database and analysis system [4].

**Figure 1:**
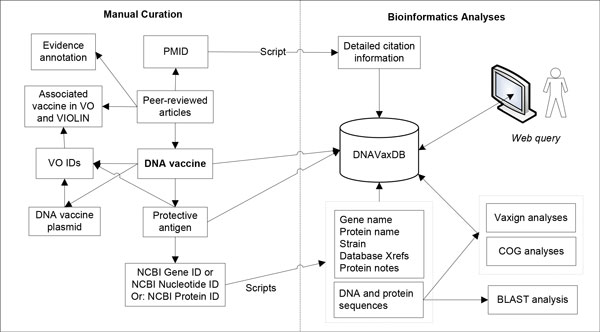
**DNAVaxDB workflow and system design**. Data, including vaccine information and evidence from peer-reviewed publications, is manually curated into DNAVaxDB. Gene IDs, Protein IDs, or Nucleotide IDs are retrieved from NCBI and used by an internal script to gather detailed information about each protective antigen, which is then stored in DNAVaxDB. DNA and protein sequences can be used for BLAST, COG, and Vaxign analyses, and the latter two are stored in DNAVaxDB. A PubMed ID (PMID) from the relevant paper is also used by an internal script to gather comprehensive citation information for each study. Each vaccine and plasmid is assigned a Vaccine Ontology (VO) ID.

Figure 1 shows the DNAVaxDB workflow and system design. For each vaccine, the DNAVaxDB database contains at minimum the following information: (1) General DNA vaccine information, including name, host animal used as model, immunization route, vector, antigen name, stage of vaccine development (*e.g*. research, clinical trial, or licensed), and efficacy. Demonstration of protection through a challenge experiment or a protective level of antibodies is necessary for inclusion in DNAVaxDB. This information is manually curated to emphasize the details of the paper, particularly the protection experiments. Antigens inserted into the vector are searched for in the NCBI Gene, Protein, and Nucleotide databases. If found, they are added to VIOLIN by using the Gene, Protein, or Nucleotide ID and an internal script to retrieve the information in NCBI, labeled as a "protective antigen," and linked to the DNA vaccine in VIOLIN. (2) DNA vaccine plasmid information. For each plasmid, the database includes name, Vaccine Ontology ID [6,7], and reference, and, if available, manufacturer, promoter, antibiotics resistance gene, and length. (3) Comprehensive citation information for each vaccine such as title, authors, journal, issue, volume, year, and pages. These references are usually found in PubMed [8], and the information found in VIOLIN is retrieved from PubMed using the PubMed ID (*i.e*., PMID) and an internal script. Each vaccine and plasmid is assigned a Vaccine Ontology (VO) identifier, linking the two databases.

### Semi-automatic annotation of vaccine information

**Figure 2:**
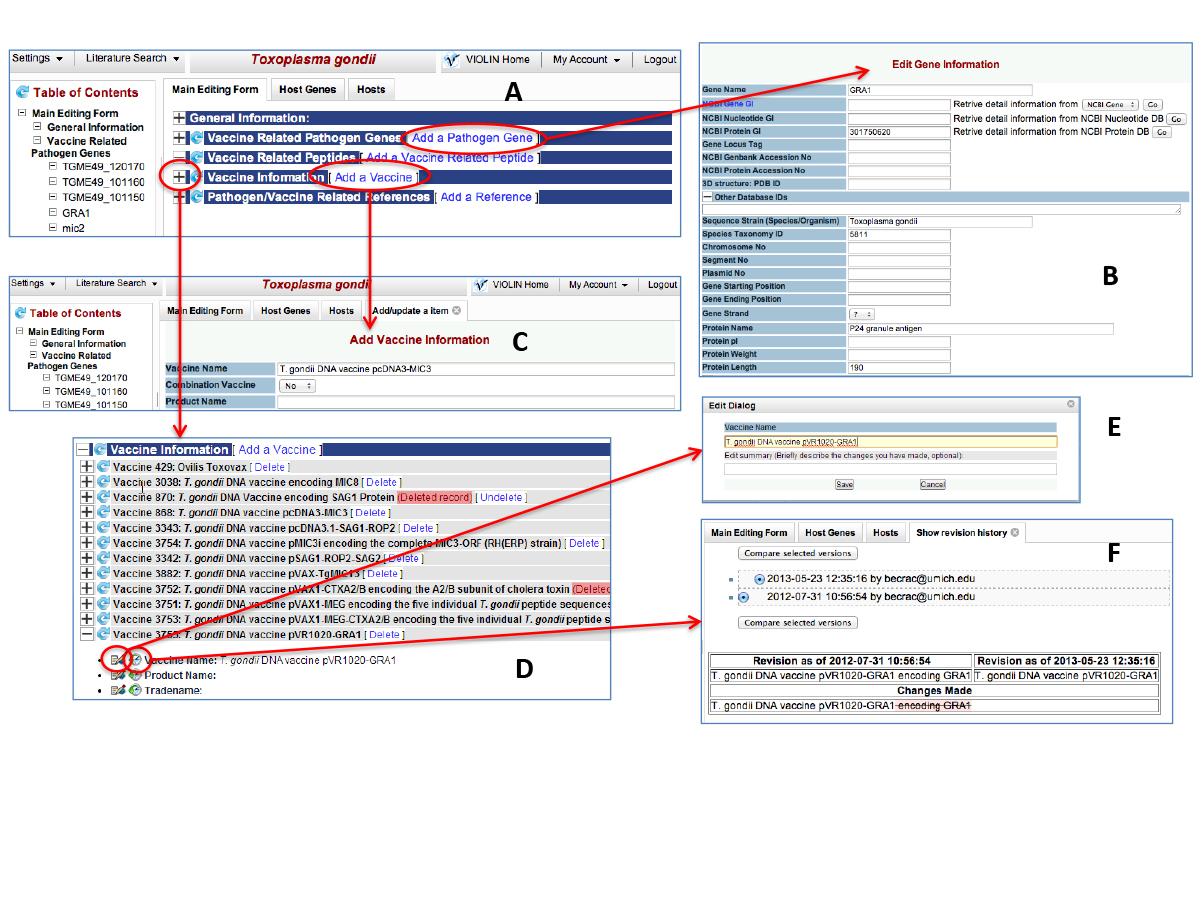
**Submission and review processes of DNA vaccines into DNAVaxDB**. A) The overall view of the top part of the DNAVaxDB data submission and review page. B) Gene submission form. Once an NCBI Gene, Protein, or Nucleotide ID is entered, an internal script is used to retrieve information such as Taxonomy ID, protein name, and DNA and protein sequences from NCBI to create a new gene entry in VIOLIN. C) Vaccine addition page. Information such as vaccine name, plasmid and antigen information, efficacy, and references can be added on this page. D) Vaccine information section includes all vaccines curated for this pathogen. Any specific vaccine can be further examined and edited. E) Edit Dialog box. Curators and reviewers can use this tool to edit a specific part of a vaccine (e.g., vaccine name). A reference may also be added for many items of information (e.g., vaccine efficacy). F) Show Revision History page. Curators and reviewers can use this tool to see what changes have been made and compare page edits.

A semi-automatic DNAVaxDB annotation system is developed for DNA vaccine and related data curation and analysis by modifying an in-house web-based literature mining and curation system called Limix [4,9,10] (Figure 2). This system includes two parts: vaccine and protective antigen data curation based on the existing VIOLIN curation system [4], and a newly developed database specifically for DNA vaccine plasmids. The web-based DNAVaxDB annotation system provides many interactive features for curators to easily obtain information. For example, once an NCBI Gene ID is entered, the system can automatically retrieve all the gene and protein information (*e.g*., DNA and protein sequences, names, functions) from the NCBI Gene database [11] (Figure 2A and 2B). Similarly, a PMID is sufficient for the system to retrieve all reference information. The curation system also allows a data curator to search literature, copy and edit text, and submit data to database (Figure 2C-2E). After a curator submits data, a domain expert will serve as the data reviewer to review, edit, and approve the curated data through a user-friendly web interface. Immediately after the approval by a reviewer, the curated data submitted will be posted publicly. The DNAVaxDB annotation also stores all the history changes and traces the detailed changes made to each record (Figure 2F).

### Analysis of protective antigens used in DNA vaccine development

For each protective antigen used in DNA vaccine development, the Vaxign software program was used for analysis of subcellular localization and adhesin probability [12,13]. The analysis of the Clusters of Orthologous Groups (COG) for each protective antigen was also performed.

A DNAVaxDB BLAST search program has been developed to support sequence similarity search on our customized BLAST library that contains over 370 protective antigens used in DNA vaccine development.

### DNAVaxDB data query, visualization, and analysis

Three user-friendly web interfaces were developed for users to separately query DNA vaccines, protective antigens, and DNA vaccine plasmids. The three parts of queries are also interlinked. For example, the display of the query results of a DNA vaccine will typically include the plasmid and protective antigen(s) used, and the name or IDs of these plasmid and antigens can then be linked to other web pages specifically for the plasmid and antigen.

To perform some systematic bioinformatics analyses, sometimes we developed MySQL scripts that could be used to directly interact with the backend MySQL database. This activity was often performed to quickly categorize and compare various statistics and characteristics of the DNA vaccines, plasmids, and antigens.

### DNAVaxDB data exchange, transfer, and download

The information of the DNA vaccines, protective antigens, and DNA vaccine plasmids from DNAVaxDB is stored in Vaccine Ontology (VO; http://www.violinet.org/vaccineontology) [6]. The VO is developed using the Web Ontology Language (OWL) that can be automatically processed via software programs [14].

## Results

### DNAVaxDB statistics

As of September 3, 2013, DNAVaxDB has included 417 DNA vaccines. Each of these DNA vaccines was experimentally verified to be protective or therapeutic with at least one laboratory animal model. In total, these DNA vaccines use 141 DNA vaccine plasmids, and 375 protective antigens have been curated for use in DNA vaccines. Occasionally, more than one antigen is used in a single DNA vaccine. These vaccines are developed against 99 infectious and non-infectious diseases (including arthritis, cancer, and diabetes). Table 1 lists infectious and non-infectious diseases for which DNAVaxDB stores 10 or more vaccines. Some protective antigens are orthologs from different strains. For example, *Brucella *has 15 protective antigens listed. However, only 10 of them are unique; the other 5 are orthologs of some of the 10 genes from different *Brucella *strains.

**Table 1:** Diseases with 10 or more DNA vaccines in DNAVaxDB

	Number of DNA vaccines	Number of protective antigens used	Number of DNA vaccine plasmids used
**G+ bacteria**			

*Bacillus anthracis*	15	11 (5)*	10

*Mycobacterium tuberculosis*	10	8 (8)	7

**G- bacteria**			

*Brucella *spp.	15	15 (10)	7

**Viruses**			

Dengue virus	13	8 (3)	6

Herpes simplex virus types 1 and 2	19	11 (6)	13

Human immunodeficiency virus	12	34 (10)	10

Influenza virus	46	27 (5)	21

**Parasite**			

*Plasmodium *spp.	10	12 (8)	9

*Trypanosoma cruzi*	10	8 (7)	7

**Non-infectious disease**			

Cancer	34	34 (29)	21

*The number inside the parentheses () represents non-ortholog genes

### Analysis of DNAVaxDB vaccine DNA plasmids

The most popular plasmids found in DNAVaxDB were analyzed (Table 2). Among the most commonly used plasmids were pcDNA3.1, pcDNA3, pVAX1, pVR1012, and pCI. Based on the information available on the manufacturers' websites, peer-reviewed articles, and other reliable sources, certain patterns were established among these popularly used plasmids. The first pattern found was the common use of the human cytomegalovirus/immediate-early (CMV) promoter among almost all the plasmids. The two popular plasmids that did not use this promoter, pCAGGS and pCAGGSP7, utilized the AG/CMV-IE/lac promoter (http://www.addgene.org/vector-database/2042/). The CMV promoter elicits higher expression levels than other promoters, and therefore is commonly used.

**Table 2:** Plasmids in DNAVaxDB with five or more associated vaccines

*Plasmid*	*VO ID*	*No. of Vaccines*	*No. of G-/+ bacterial vaccines*	*No. of viral vaccines*	*No. of parasitic vaccines*	*No. of fungal vaccines*	*No. of other vaccines*
pcDNA3.1	VO_0000158	49	15/4	16	7	0	7
pcDNA3	VO_0000132	40	4/1	16	11	0	8
pVAX1	VO_0000024	24	1/2	8	10	0	3
pVR1012	VO_0000334	19	0/4	7	5	1	2
pCI	VO_0000212	16	2/4	9	0	0	1
pCI-neo	VO_0000214	12	6/1	5	0	0	0
pWRG7077	VO_0000346	11	0/1	9	0	0	1
pJW4303	VO_0000276	10	3/5	1	0	0	1
pCMVi-UB	VO_0005027	10	10/0	0	0	0	0
pCAGGS	VO_0000099	10	0/0	10	0	0	0
pIRES	VO_0000262	9	0/0	9	0	0	0
pCAGGSP7	VO_0005000	8	0/0	8	0	0	0
pVAX	VO_0000019	8	0/0	6	0	0	2
pCMV	VO_0000215	7	0/0	7	0	0	0

Secondly, several commonly used plasmids utilized an SV40 polyadenylation terminator. This type of terminator is commonly used to reduce additional peptide expression or alteration of the expressed peptide [15].

Thirdly, some plasmids have been more frequently used for development of DNA vaccines against one type of pathogens than the others (Table 2). For example, pCAGGS has been used for development of 10 successful viral DNA vaccines; however, this plasmid has not been used for any other types of DNA vaccines stored in DNAVaxDB. Similarly, 10 Gram-negative bacterial DNA vaccines use the plasmid pCMVi-UB, and this plasmid has not been used for DNA vaccines against any Gram-positive bacteria or other types of pathogens (Table 2).

Finally, most of the commonly used plasmids with information available about antibiotic resistance markers employed an ampicillin marker. This is a noteworthy observation, since there is potential hypersensitivity to ampicillin in some patients, and therefore these plasmids would not be acceptable for human use. Kanamycin is a more acceptable marker for human use [15]. Further research is necessary to determine what makes these plasmids so effective in lab animals and what can be done to modify them for human use, such as replacing the ampicillin resistance marker with a kanamycin marker and finding new ways to further increase antigen expression.

### Analysis of bacterial DNA vaccines in DNAVaxDB

In total, there are 95 protective bacterial antigens used in DNA vaccines stored in DNAVaxDB. These protective antigens are also cross-described in the VIOLIN Protegen database [16]. It has been reported that most protective antigens for vaccine development are secreted extracellular bacterial proteins, cell surface proteins, or adhesins [16]. Therefore, one important aspect of this study is to analyze these DNA vaccine antigens in terms of their subcellular localization and adhesin probability and compare these with the general patterns when all protective antigens for any types of bacterial vaccines are considered [16].

As shown in Table 3, of the 28 Gram-positive bacterial protective antigens, 18 (64%) have extracellular localization, and 3 (11%) are cell wall proteins. The remaining antigens are distributed between cytoplasmic region (2 or 7%) and cytoplasmic membrane (5 or 18%). In addition, 11 antigens (39%) are adhesins or adhesin-like proteins. Therefore, extracellular and adhesin antigens are superior targets for Gram-positive DNA vaccine construction.

**Table 3:** Subcellular localization and adhesin probability of bacterial protective antigens

	Gram + bacteria	Gram - bacteria	*Chlamydophila*
Total	28	67	14
Extracellular	18 (64%)	11 (16%)	1
Cell wall	3 (11%)	N/A	N/A
Unknown	0	18 (27%)	2 (15%)
Cytoplasmic	2 (7%)	12 (18%)	6 (46%)
Cytoplasmic Membrane	5 (18%)	2 (3%)	2 (15%)
Periplasmic	N/A	4 (6%)	0
Outer Membrane	N/A	20 (30%)	3 (23%)
Average Adhesin Probability (# antigens with > 0.51)	0.43 (11, 39%)	0.40 (20, 30%)	0.26 (0)

Of the 67 Gram-negative bacterial protective antigens, the extracellular and outer membrane locations are among the major subcellular regions with 11 and 20 protective antigens, respectively (16% and 30%). There are 12 cytoplasmic protein antigens (18%). The numbers of cytoplasmic membrane and periplasmic proteins are small. However, there are 18 antigens with unpredicted subcellular localization. Our study also found that 20 antigens (30%) used in Gram-negative bacterial DNA vaccines are adhesin or adhesin-like proteins (Table 3). In summary, we found that the extracellular or surface or adhesin proteins preferred targets for Gram-negative DNA vaccine construction; meanwhile, cytoplasmic proteins also form a group of frequently used antigens in this type of DNA vaccines.

Although the extracellular, cell surface, and adhesin proteins are preferred bacterial DNA vaccine antigens, the data shown in DNA vaccines against *Chlamydophila *provides a different view (Table 3 and 4). Fourteen unique protective *Chlamydophila *antigens from the Gram-negative bacterial pathogen were used for development of experimentally verified DNA vaccines. This species did not have any antigens that were repeatedly used with different adjuvants or in different combination with other antigens. These antigens have many different functions, and in total there are 7 different COG categories. Among the 14 protective *Chlamydophila *antigens used, 6 were cytoplasmic, 2 were at the cytoplasmic membrane, 3 were at the outer membrane, 1 was extracellular, and 2 were unknown. However, the two protective antigens with unknown localizations are believed to be localized to the cytoplasm; GatC is part of a larger complex also including GatA and GatB, which are both localized to the cytoplasm, and Ssb is a DNA binding protein and DNA is localized to the cytoplasm. Therefore, different from the common pattern of high percentage of extracellular and outer membrane antigens, the majority of protective antigens used for *Chlamydophila *DNA vaccines are cytoplasmic proteins.

**Table 4:** Antigens in DNAVaxDB for *Chlamydophila *spp.

*Antigen*	*Protein ID*	Species	*Localization (probability)*	*COG*	*PMID*
CAB049	62184696	*C. abortus*	Cytoplasmic membrane (1)	Cell wall/membrane biogenesis	15811648
CAB613	62185225	*C. abortus*	Cytoplasmic (0.997)	Amino acid transport and metabolism	15811648
DnaK	26006347	*C. abortus*	Cytoplasmic (0.997)	N/A	12056482
DnaX	62148024	*C. abortus*	Cytoplasmic (0.997)	N/A	15811648
GatA	62184918	*C. abortus*	Cytoplasmic (0.997)	Translation, ribosomal structure and biogenesis	15811648
GatB	62147986	*C. abortus*	Cytoplasmic (0.896)	N/A	15811648
GatC	62184917	*C. abortus*	Unknown (0.2)	Translation, ribosomal structure and biogenesis	15811648
OmlA	62184824	*C. abortus*	Outer membrane (1)	N/A	15811648
Pomp90A	187438939	*C. abortus*	Extracellular (0.946)	N/A	15811648
Omp1	40601	*C. abortus*	Outer membrane (1)	N/A	15811648
FabD	15618217	*C. pneumoniae*	Cytoplasmic (0.926)	Lipid transport and metabolism	16434129
PknD	161353778	*C. pneumoniae*	Cytoplasmic (0.788)	Signal transduction; transcription; replication, recombination, and repair	16434129
Ssb	15618301	*C. pneumoniae*	Unknown (0.2)	Replication, recombination, and repair	16434129
OmpA	94467392	*C. psittaci*	Outer membrane (0.992)	N/A	20199760

### Analysis of viral DNA vaccines in DNAVaxDB

In total, DNAVaxDB contains over 200 DNA vaccines against over 40 viruses. In order to get a closer look at viral DNA vaccines, we chose to analyze human immunodeficiency virus (HIV) and the related diseases simian-human immunodeficiency virus (SHIV) and simian immunodeficiency virus (SIV). HIV is a prevalent problem worldwide. The SHIV and SIV models have been frequently used for HIV vaccine studies. Information about the HIV, SHIV, and SIV vaccines can be found in Table 5. HIV-1 and SHIV89.6P were commonly used strains for challenge, but there were no strains that were used the majority of the time. Overall, the HIV, SHIV, and SIV DNA vaccines found in DNAVaxDB are diverse. Many aspects of the HIV, SHIV, and SIV DNA vaccines do not follow any particular pattern. All fourteen of the vaccines are quite unique and utilize many different techniques in order to obtain protection.

**Table 5:** HIV, SHIV, and SIV DNA vaccines

#	DNA Vaccine	Antigen name(s)	Challenge: HIV/SHIV strain	DNA plasmid	Vaccination regimen	PMID (year)
**DNA vaccine alone**						

1	HIV DNA vaccine VlJns-tPA-gp120	gp120	HIV-1	VlJns	DNA vaccine alone	9127013 (1997)

2	HIV-2 DNA vaccine	gag, nef, tat, env	HIV-2UC2/9429	pND-14	DNA vaccine with GM-CSF and B7-2 adjuvants	15149785 (2004)

3	SIV DNA vaccine encoding SIV core protein	Core protein	SHIV89.6P	pCSIVgag	DNA vaccine with IL-15 adjuvant	18000037 (2007)

4	DNA vaccine expressing multiple HIV epitopes	rev, nef, tat, gag, env, pol	HIV-1	Auxo-GTU vector system	DNA vaccine alone	16359234 (2005)

5	HIV DNA vaccine pHIS-SHIV-B	env, gag, pol, tat, rev, vpu	SHIV(mn229)	pHIS-64	DNA vaccine alone	15564490 (2004)

6	HIV DNA vaccine pCMN160	env, rev, gag-pol	HIV-1 SF2	pCMN160	DNA vaccine alone	9142121 (1997)

**DNA vaccine priming and other vaccine boosting**						

7	HIV DNA vaccine SIVmac239 Gag-Pol-Nef and mock Env with rAd5 boost	gag, pol, nef, env	SHIV89.6P	pVR1012	DNA vaccine prime, adenovirus boost	15613305 (2005)

8	HIV DNA and adenoviral vector Ad5 expressing SIV gag protein	gag	SHIV 89.6P16	pV1R	DNA prime, adenovirus boost	11797011 (2002)

9	HIV DNA vaccine SHIV-89.6 DNA (DNA/89.6)	gag, pol, env, tat, rev, vif, vpr, vpu	SHIV-89.6P	pGA	DNA vaccine with GM-CSF adjuvant prime, MVA boost	16740288 (2006)

10	DNA and poxvirus priming-boosting SHIV vaccine	env, gag	SHIV-89.6P	pV1R	DNA prime with IL-2/Ig adjuvant, poxvirus boost	15258286 (2004)

11	HIV priming with DNA vaccine expressing HIV gp160 protein and boosted with Ad5/35 vector	env, rev	HIV-1	pCAGGS	DNA prime, adenovirus boost	16079886 (2005)

12	SHIV DNA vaccine encoding env and gag	env (4 from 3 clades*), gag	SHIV-Ba-L	pSW3891	DNA vaccine prime, protein boost	16460776 (2006)

13	SHIV(Ba-L) DNA vaccine encoding env and gag	env (5 from 4 clades), gag	SHIV-Ba-L	pSW3891	DNA vaccine prime, protein boost	16128917 (2005)

14	SIV DNA vaccine pVacc4 encoding env from mac239	env	SHIV89.6P	pVacc4	DNA vaccine with IL-2/Ig adjuvant prime, MVA boost	15004179 (2004)

*The polyvalent DNA vaccines encoded four env genes from four four primary HIV-1 isolates in three clades: 92US715.6 (clade B), Ba-L (clade B), 96ZM651 (clade C), and 93TH976.17 (clade E).

There were many different antigens used, the most common being *env *and *gag*. Env is an envelope protein, which is synthesized as gp160 and later cleaved into gp120 and gp41 [17]. Gag functions in the construction of virus-like particles (VLPs) [18]. Frequently more than one antigen has been used for HIV vaccine development (Table 5).

Only two plasmids were repeatedly used for at least two vaccines: pV1R and pSW3891. Although pcDNA3.1 is the most common DNA vaccine plasmid used in DNA vaccine development according to the statistics in DNAVaxDB (Table 2), this plasmid is not frequently used in HIV vaccine development. A few studies used pcDNA3.1 for HIV vaccine development, but protection was not evaluated in these investigations. For example, Du and colleagues built two vaccines, pcDNA-Vif and pcDNA-LIGHT, both based on pcDNA3.1. They then measured antibodies and T-cell responses [19]. However, since a protection study was not done, we could not include this paper in DNAVaxDB.

One pattern that did stand out was the division between prime-boost vaccines that employed the DNA vaccine as a prime and DNA vaccination alone. Eight of the vaccines in DNAVaxDB are prime-boost vaccines, and six are DNA vaccines alone. Five out of the 14 HIV DNA vaccines are also mixed with adjuvants to boost host immune responses. After analysis of the two groups, it was discovered that most of the prime-boost vaccines demonstrated high or complete protection of all challenged animals. Looking at the dates of each study, it's also clear that the prime-boost studies were more recent while the older studies were usually DNA vaccination alone.

### Analysis of parasite DNA vaccines in DNAVaxDB

DNAVaxDB contains 61 DNA vaccines against 16 protozoan pathogens. The two pathogens with the most vaccines were *Plasmodium *spp. and *Toxoplasma gondii*, as seen in Table 1. These two pathogens also had the most protective antigens out of all parasites. Analysis of *Plasmodium *vaccines showed a wide range of vaccination methods. Variety was seen most commonly in immunization route (i.e. gene gun, intramuscular, etc.) and vaccination regimen (i.e. with or without an adjuvant, with or without boosting). However, as with all vaccines in DNAVaxDB, all these different methods proved efficacious and provided protection, demonstrating that there are multiple ways to stimulate a host's immune response via DNA vaccine.

### Analysis of cancer DNA vaccines in DNAVaxDB

Cancer vaccines found in DNAVaxDB were analyzed, as cancer is the major non-infectious disease found in the database. Of the 34 cancer protective antigens, 8 were localized to the cytoplasmic membrane, 3 to the cytoplasm, 1 to the outer membrane, 2 are extracellular, and 20 have an unknown location. Eliminating those with an unknown location, 11 of the 14 protective antigens have locations near or on the outside of the cell. This makes for easier access for antibodies. Another noteworthy observation is that nearly all the vaccines stored in DNAVaxDB are used for the prevention of tumors rather than the treatment of existing tumors. While cancer prevention is important, treatment is more clinically significant [20]. DNA vaccines for cancer treatment need to be able to elicit a tumor-specific response to kill tumor cells and control the growth of new cells. The vaccines that have been discovered for cancer treatment thus far are effective using experimental models. However, in most cases, clinical trials are needed to evaluate the possibilities of using these DNA vaccines to confer protective immunity in humans.

### DNAVaxDB data query, display, and data sharing

**Figure 3:**
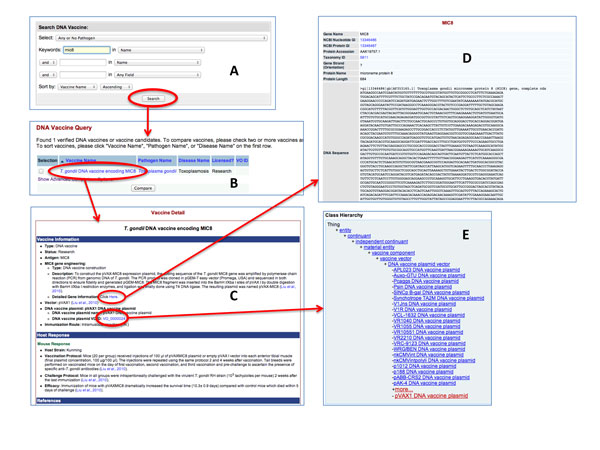
**An example of searching a vaccine and its related information in DNAVaxDB**. (A) The vaccine name "mic8" is queried in DNAVaxDB. (B) The resulting vaccine in DNAVaxDB. (C) The DNAVaxDB page obtained by clicking on "*T gondii *DNA vaccine encoding MIC8" in (B). The DNAVaxDB page for the vaccine has information about the vaccine (protective antigen inserted, vector, host response, etc.). (D) The gene information page obtained by clicking on the "Detailed Gene Information Here" link from (C). This gives more information about the protective antigen used in the vaccine. (E) The Ontobee [40] page detailing the hierarchy of VO and showing the place of pVAX1, the plasmid used in this vaccine. This page is obtained by clicking on the VO ID in (C). The plasmid name can also be clicked to access a plasmid-specific page (data not shown).

All information stored in DNAVaxDB is publically available for download and exchange as described in the Materials and Methods section. The curated DNAVaxDB data can be proficiently queried by vaccine, plasmid, or protective antigen as seen in Figure 3.

The Vaccine Ontology (VO) is a community-based ontology that represents the vaccines and vaccine-related terms and the relations among these terms [7,21]. VO is developed using the machine-processable Web Ontology Language (OWL) format [22]. All the DNA vaccines, DNA vaccine plasmids, and protective antigens used in these DNA vaccines, and the relations among these terms, have been logically represented in VO. The inclusion of these data in VO allows software programs to easily process the data and use them for further application. In addition, the vaccine information in the Vaccine Ontology (VO) has also been used to integrate the data in the DNAVaxDB web project design. For example, the VO information is queried using internal SPARQL [23] scripts against a Resource Description Framework (RDF) triple store [24] that stores the ontology contents. The VO IDs of different vaccines are used as the glue that links the vaccines in the DNAVaxDB database and the general VIOLIN vaccine database.

## Discussion

To the best of our knowledge, DNAVaxDB is the first web-based, publically available database and analysis system that targets the curation and analysis of DNA vaccines, related DNA vaccine plasmids, and protective antigens. DNAVaxDB contains manually-curated DNA vaccines that have been used in the prevention and treatment of a wide variety of infectious diseases, as well as diseases such as cancer. Analysis of DNAVaxDB data has allowed us to reveal specific patterns from the experimentally verified DNA vaccines, plasmids, and protective antigens. These results support better understanding of protective DNA vaccine mechanisms and facilitate rational DNA vaccine design.

A few challenges have been identified throughout the DNA vaccine curation process. One challenge is that many DNA vaccines have been developed, but very few offer significant protection against challenge. Finding vaccines that offered robust protection in animal models could be difficult at times. To make DNAVaxDB valuable, we insisted that all DNA vaccines stored in the database were experimentally verified to induce protection in at least one laboratory animal model. Accuracy will not only provide more precise analysis, but also make DNAVaxDB a more useful tool for researchers.

In most cases, our studies with protective antigens collected in DNAVaxDB are consistent with previous finding that the bacterial surface or extracellular proteins are often good vaccine candidates [10,16]. We have previously identified another group of microbial genes called "virmugens", which is coined to represent a gene that encodes for a pathogen virulence factor and has been proven feasible in at least one animal model to make a live attenuated vaccine after this gene is knocked out [25]. It has been known that virulence or pathogenic factors can often be used as protective antigens [26]. Our study also found that some virmugens, such as herpes simplex virus 1 gD [27,28], *Brucella melitensis *Omp25 [29,30], and influenza virus M2 [31,32], are used for DNA vaccine development and function as protective antigens. More specific studies should be performed to investigate the relationships among general protective antigens, protective antigens used in DNA vaccine development, and virmugens.

Although many bacterial DNA vaccines use surface or extracellular proteins as protective antigens, surprisingly almost the half of the *Chlamydophila *protective antigens used in DNA vaccines are cytoplasmic, Cytoplasmic inner proteins are typically not accessible to antibodies. It suggests that other immune response mechanism(s), such as cytolytic T lymphocytes (CTL) reactivity inducible by both inner and outer proteins, are most likely responding to these antigens [1]. However, antibodies are an important immune response, and the inability to generate such an antibody response would seem to be detrimental to a vaccine. Several of these candidates came from one paper, who also discovered that the outer membrane proteins are not always protective either [33]. More work needs to be done on this pathogen as well as other pathogens to better understand the protective vaccine mechanisms associated with different types of antigens.

As demonstrated in the patterns identified from the HIV, SHIV, and SIV DNA vaccine research, the prime-boost vaccination with DNA priming has been of particular interest recently due to its effectiveness. This vaccination regimen is able to elicit a T cell response that is focused on the antigens encoded in the vaccine. This allows for a focused and potent immune response [34]. The prime-boost method is also cost-effective; DNA vaccines alone are fairly inexpensive to manufacture. However, DNA vaccines must be administered in high doses to be effective alone, which negates their cost effectiveness advantage. By using a DNA vaccine as a priming vaccine, which can be administered in much smaller quantities than if the DNA vaccine was used alone, it will result in a cost advantage. Further, some boosting vaccines, such as protein vaccines, can be expensive, so by using DNA vaccines as a priming vaccine instead of a protein vaccine as both a priming and a boosting vaccine, it provides further savings [35]. Prime-boost vaccines in DNAVaxDB also used a variety of vaccines for the boost. Three of the eight HIV DNA vaccines utilized adenovirus vectors (Table 5). These vectors are popular due to their ability to elicit strong T cell responses, and several different serotypes of adenoviruses have been explored as potential vectors. However, several other vectors, such as poxviruses and modified vaccinia virus Ankara (MVA), have been used to generate recombinant vector vaccines for boosting, and the administration of protein boost vaccines has also been frequently used for boosting host B cell immune responses [34,36-39]. The frequent use of the prime-boost regimen strategy is evident in DNAVaxDB, as the database contains many prime-boost DNA vaccines with different types of vaccines for boosting.

The DNAVaxDB database and analysis system has many applications, with one being the ability to support rational vaccine design. Since DNAVaxDB stores both plasmids and protective antigens used in DNA vaccines, this information can be used to mix and match the components to create the safest and most efficacious vaccines possible. Further, DNA vaccines can be designed based on successful plasmid and protective antigen combinations in other pathogens. We plan to develop new software capabilities in the future to support rational DNA vaccine design.

DNAVaxDB is slated to become a vital source of DNA vaccine information and will support researchers in the areas of immunology, vaccinology, and microbiology. As a central and vital source of DNA vaccines, DNAVaxDB is a timely repository and is expected to have a significant impact for vaccine research and development.

## List of abbreviations

CMV: Cytomegalovirus; COG: Clusters of Orthologous Groups; CTL: Cytotoxic T lymphocyte; DNA: Deoxyribonucleic acid; HIV: Human immunodeficiency virus; MVA: Modified vaccinia virus Ankara; NCBI: National Center for Biotechnology Information; PMID: PubMed identification number; RDF: Resource Description Framework; SIV: Simian immunodeficiency virus; SHIV: Simian-human immunodeficiency virus; OWL: Web Ontology Language; VIOLIN: Vaccine Investigation and Online Information Network; VO: Vaccine Ontology

## Competing interests

The authors declare that they have no competing interests.

## Authors' contributions

RR: Data curation and review, data analysis, and manuscript writing. XL, MP: Data curation. ZX: Data processing, software programming, and database administrator. YH: Project design, result analysis and interpretation, and manuscript writing. All authors approved the paper publication.

## Declaration statement

The publication funding came from NIH-NIAID grant R01AI081062.

This article has been published as part of BMC Bioinformatics Volume 15 Supplement 4, 2014: Selected articles on Computational Vaccinology 2013. The full contents of the supplement are available online at http://www.biomedcentral.com/bmcbioinformatics/supplements/15/S4.
